# Transcending Self Therapy-Virtual Reality (TST-VR) and Substance Use Disorder (SUD) Treatment Completion in Veterans

**DOI:** 10.1089/jmxr.2024.0025

**Published:** 2024-10-18

**Authors:** Anna M. Wiese, Jarrod Reisweber, Mark Lambert, James M. Bjork

**Affiliations:** 1 Department of Psychology, Virginia Commonwealth University, Richmond, VA, USA.; 2 Central Virginia VA Health Care System, Richmond, VA, USA.; 3 Lighthouse XR, Richmond, VA, USA.; 4 VISN 6 Mental Illness Research, Education, and Clinical Center, Richmond, VA, USA.

**Keywords:** substance use disorder, veterans, virtual reality, treatment outcome

## Abstract

Transcending Self Therapy (TST) is an integrative Cognitive Behavioral Therapy (CBT) for substance use disorders (SUD). Virtual reality (VR)-based enhancement of treatment for mental health disorders holds potential as an innovative and immersive adjunct to standards of care. However, outside of cue-conditioning applications, how VR may assist SUD treatment is not well understood. The aim of this study was to examine the association between engagement with a VR version of TST and residential SUD treatment completion. In a program evaluation, 51 consecutive military veterans admitted to a residential SUD treatment program were afforded access to VR equipment programmed to enable *ad libitum* exploration of a virtual wooded retreat with stations that reinforced psychoeducation concepts of TST, meditation, and other therapeutic activities. Administrative records were reviewed, and treatment completion data were collected for veterans who opted to receive a TST-VR headset (*n* = 36) and those who did not (*n* = 15). Engagement with the VR gear and program was related to treatment completion. Veterans who opted to accept the VR program and related training in its use were significantly more likely to complete treatment than those who did not (86.1% vs 60.0%; *p* = 0.039). These data suggest that either VR-based reinforcement of TST concepts improved the treatment experience or motivation for change, and/or that individuals with poor general treatment motivation are unlikely to choose VR-enhancement of SUD treatment and complete treatment in general. Both possibilities have important implications for VR SUD treatment research.

## Introduction

Substance use disorders (SUD) among the veteran population represent a significant and costly public health concern.^1^ In response, Transcending Self Therapy (TST), an evidence-informed integrative Cognitive Behavioral Therapy (CBT) was developed and has demonstrated efficacy for SUD.^2^ However, the onset of the COVID-19 pandemic exacerbated existing mental health issues and substance misuse while straining clinical workforce capacity.^3^ This necessitated innovative solutions to support growing patient needs and limited provider availability,^4^ such as the implementation of psychotherapy services via telehealth, which has been shown to be efficacious. For example, TST was provided via a remote, on-screen therapist during COVID-19 social distancing, and veteran groups who received this remote therapy showed similar improvements in quality of life from pre- to posttreatment as those who were seen by an in-person therapist.^5^

**FIG. 1.**
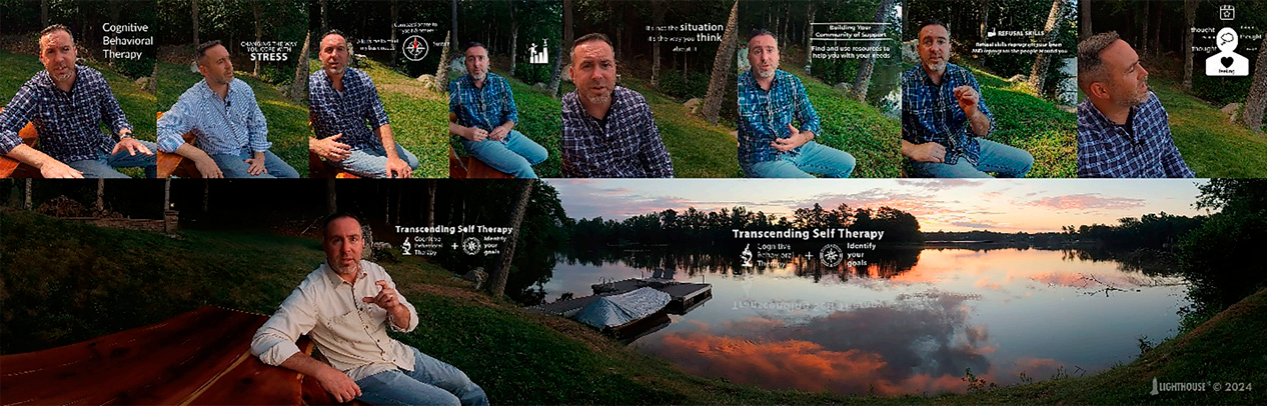
Images of the Transcending Self Therapy Virtual Reality (TST-VR) retreat platform.

One potential solution currently under increasing investigation for improving delivery of TST and CBT is virtual reality (VR). VR can be used as a mode for *ad libitum* delivery of supplemental psychoeducation lessons or reinforcement of CBT and other therapeutic concepts that are typically presented in standard group or individual therapy. With the use of VR gear, patients could view immersive videos of providers addressing important problem solving or emotion regulation concepts and even complete virtual homework (Fig. 1). With these capabilities, VR could provide a colorful and immersive adjunct to standard therapy that may improve engagement with therapeutic materials and could also improve rates of retention in treatment. This is a critical deliverable considering the strong linkage between homework completion and successful SUD treatment outcomes,^6^ as well as the higher rate of relapse in persons with against medical advice (AMA) discharges or other irregular discharges from SUD treatment.^7^

VR has been increasingly used in healthcare and SUD treatment specifically. For the purposes of SUD treatment, VR has been utilized in attempts to decouple drug cues from consumption in cue exposure-based therapies, with mixed success,^8^ as well as to deliver cognitive training in persons with disordered use of ketamine^9^ and alcohol,^10^ resulting in improved attention and other cognitive functions. Limited examples of VR as a supplement to other SUD treatment include the findings of Faraj et al.,^11^ which showed that the VR meditation sessions acutely reduced stress responses in a population with opioid use disorder undergoing methadone maintenance therapy. However, overall, VR’s use as an enhancement of psychoeducation or other therapy delivery in SUD treatment has been limited.^12^

To address this gap in the literature, TST was expanded into a VR experience that could support providers and provide additional self-guided therapeutic engagement for patients. Patient acceptance and initial assessment of the effectiveness of the resulting TST-VR program were tested among individuals in the residential substance abuse treatment program (SATP) at a Mid-Atlantic Veterans Affairs Medical Center (VAMC). Two cohorts found the apparatus comfortable and the program to be easy to use, with content helpful to their recovery and understanding of CBT.^5,13^ Given the paucity of knowledge in how VR-based delivery of psychoeducation content and activities, such as being made available to the patient as an *ad libitum* unit activity above and beyond standard of care, may improve treatment engagement and/or outcomes, further research was warranted. Thus, we conducted an administrative records review to examine the association between the individual differences in patient adoption of TST-VR and treatment outcomes, specifically the completion of residential substance use disorder treatment.

**FIG. 2.**
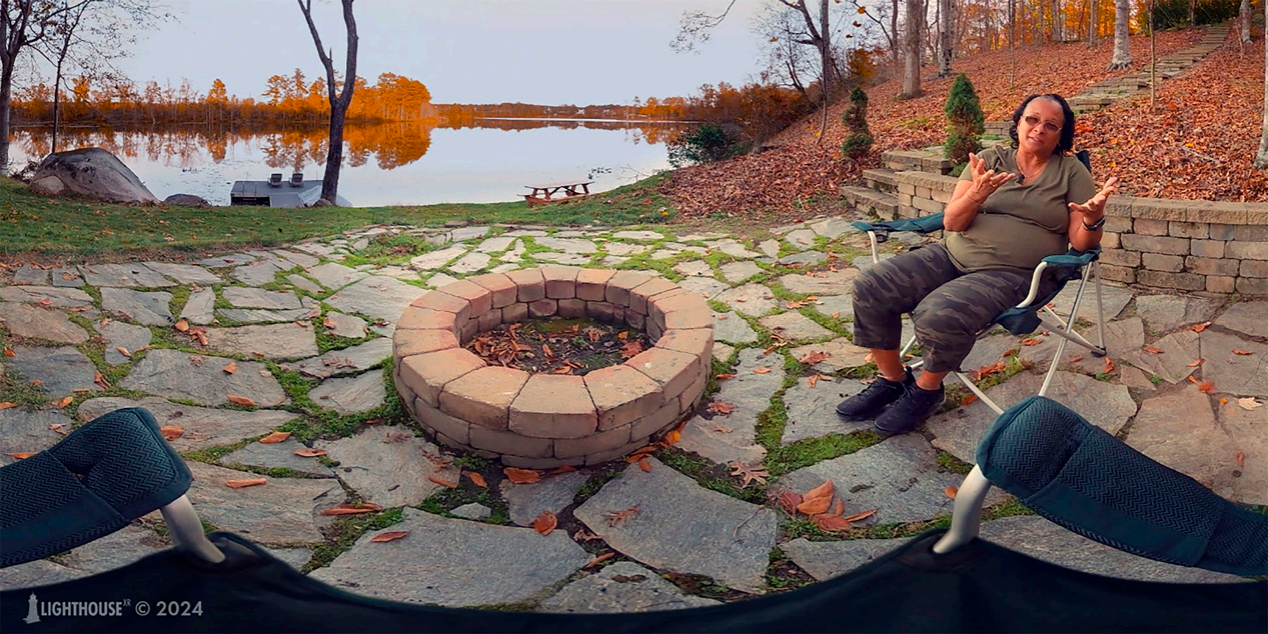
Image of experiences in the retreat platform virtual world.

In a program evaluation of VR as a standard of care adjunct to traditional group therapy, access to VR gear programmed with TST therapeutic content (along with a training session in how to use the equipment and program) was afforded to each veteran admitted to a 28-day residential treatment program who did not have a known history of seizures. We hypothesized that patients who participated in the VR program would show greater treatment completion rates than patients who did not participate in VR, for two potential reasons. First, the colorful and immersive VR experience may enhance treatment satisfaction and increase motivation for treatment and completion (Fig. 2). Second, patients with lower intrinsic motivation for treatment may be less likely to accept or participate in an optional supplemental treatment module in addition to being more likely to drop out.

## Methods

### Participants

Participants were *n* = 51 adults recruited from the 28-day residential SATP at a Mid-Atlantic VAMC from fall of 2023 through the beginning of spring of 2024 (mean age 56.1 years; 94.1% male; 5.9% female; Table 1). All newly admitted SATP patients with no known histories of seizures were introduced to the TST-VR and provided a headset if interested in utilizing the TST-VR. SATP staff trained interested and eligible patients in navigating the TST-VR headset. Data on whether patients chose to receive a headset or declined a headset were collected. Additionally, in order to examine baseline characteristics that may have affected motivation levels between those who received the TST-VR headset and those who did not, psychiatric diagnoses upon admittance to the residential treatment program were also examined via chart review. For example, SUD in the presence of a comorbid affective disorder such as depression^14^ or posttraumatic stress disorder^15^ is notoriously difficult to treat, wherein motivational decrements are increasingly appreciated as a common trans-diagnostic feature of mental illness.^16^ The local VAMC Institutional Review Board determined that this program evaluation did not constitute human subject research. Therefore, informed consent was not obtained from patients.

**Table 1.** Demographic Characteristics of Total Sample (*n* = 51)

Characteristic	*N* (%)
Age (M [SD])	56.1 [10.7]
Sex	
Male	48 (94.1)
Female	3 (5.9)
Race/Ethnicity	
Black	34 (66.7)
White	14 (27.5)
American Indian or Alaska Native	1 (2.0)
Not Provided	2 (3.9)

### The TST-VR experience

**FIG. 3.**
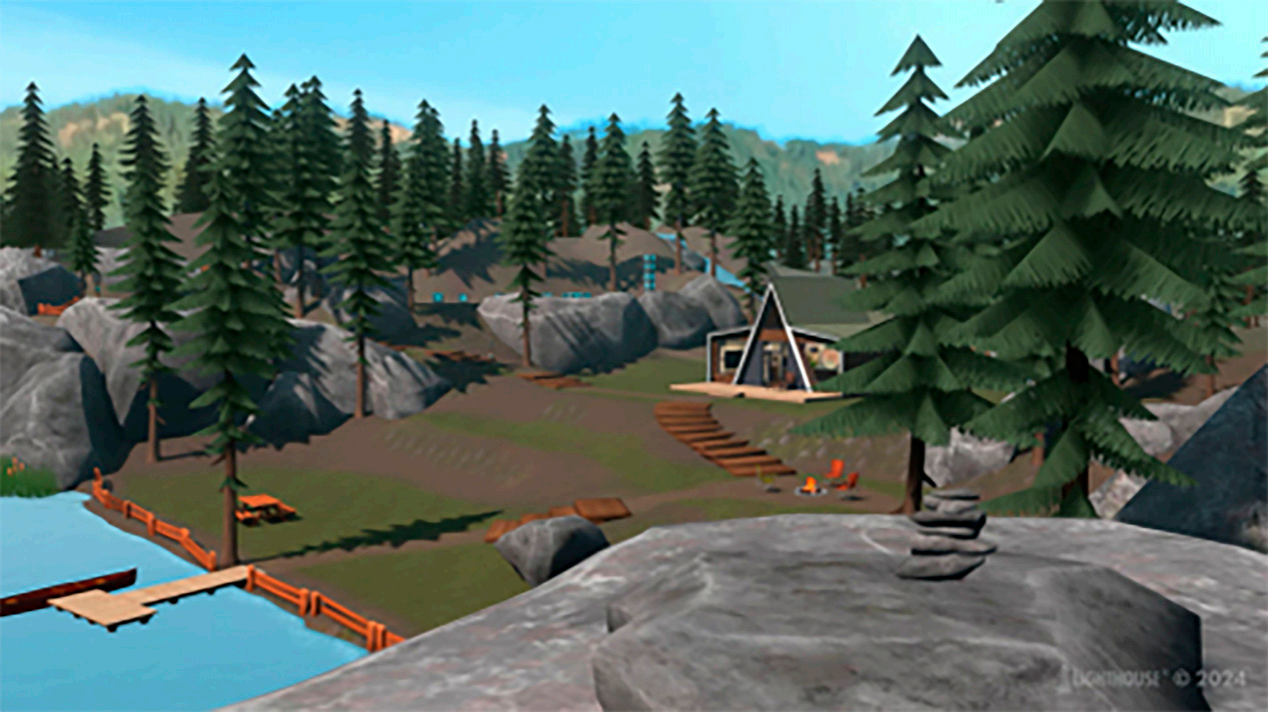
Image of the Transcending Self Therapy Virtual Reality (TST-VR) nature landscape.

**FIG. 4.**
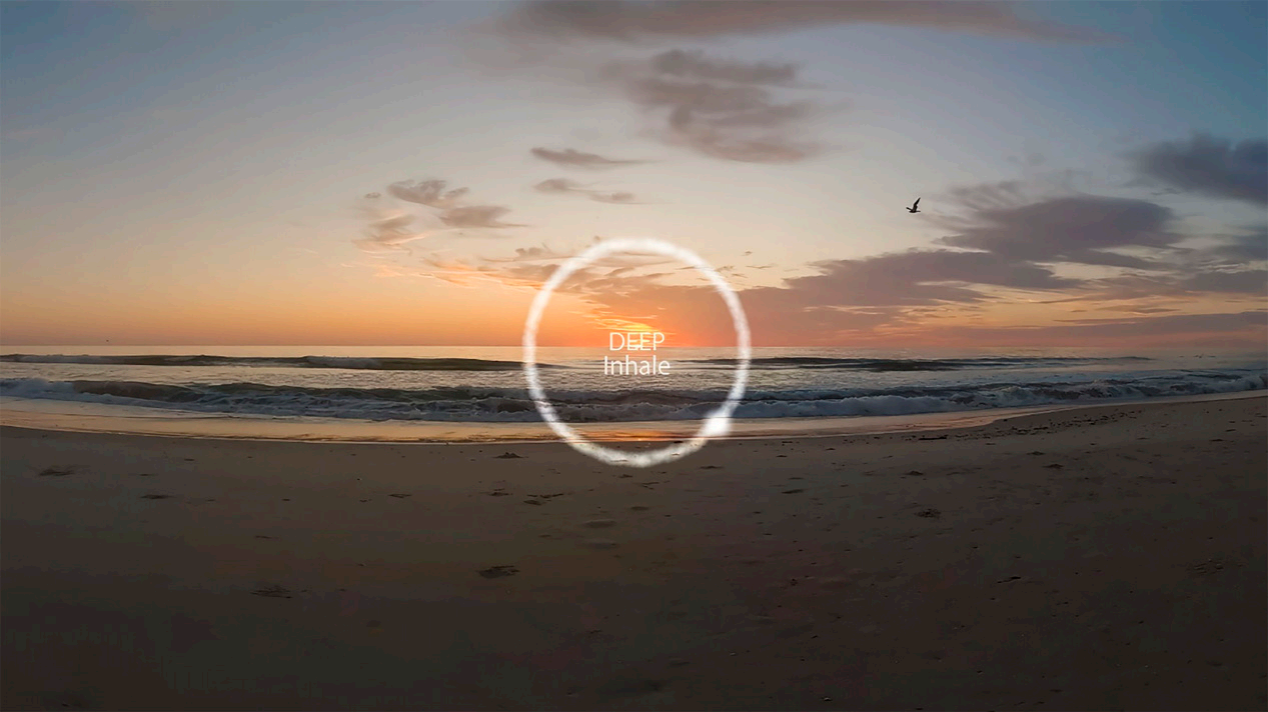
Image of the Transcending Self Therapy Virtual Reality (TST-VR) meditation activity.

TST-VR centers on a virtual “retreat” nature landscape (Fig. 3) in which patients can navigate among virtual instructional videos explaining the key concepts of TST, as well as meditation and psychoeducational videos (Fig. 4). The CBT concepts reinforced through TST-VR include cognitive conceptualizations, behavioral interventions, cognitive interventions, and obstacles to sustained recovery. TST-VR helps patients to understand their past experiences, core beliefs, and thought patterns, as well as how these topics relate to their substance use and how making changes in their thought patterns can aid in their recovery. For more specifics about the hardware and therapeutic content of TST-VR, please see Zaur et al.^13^

### Data analytic plan

Successful completion of the residential treatment program, as well as irregular discharges and AMA discharges from the program, were coded from electronic medical records and VAMC SATP program tracking logs. All veterans who were admitted to and discharged from SATP during this period of data collection, for any amount of time, were included in this analysis. Only one patient who was admitted to SATP during the data collection period was excluded from analyses due to having an irregular discharge, followed by a re-admittance and regular discharge, within a short window of time. The number of psychiatric diagnoses upon admittance to the residential treatment program was also obtained from electronic medical records. To assess potential baseline differences between those who opted to receive a VR headset and those who did not, an independent samples *t-test* was conducted to examine differences between groups in the number of psychiatric diagnoses upon admittance. Chi-square analysis was conducted to determine the association between engagement in VR [specifically, opting to be issued a VR headset (yes/no)] and successfully completing the residential SUD treatment program (yes/no).

## Results

There was no significant difference between individuals who opted to be issued a TST-VR headset (M = 3.9) and those who did not (M = 3.7) in terms of the number of psychiatric diagnoses upon admittance to treatment (*p* = 0.761). Individuals who opted to be issued a TST-VR headset were significantly more likely to successfully complete the residential SUD treatment program than those who did not receive a headset (86.1% vs. 60.0%, *p* = 0.039; Table 2).

**Table 2.** Treatment Completion by Headset Group

Variable	Received headset (*n* = 36)	Did not receive headset (*n* = 15)	*p* value
Treatment completion {[N(%)] successfully completed}	31 (86.1%)	9 (60.0%)	.039

## Discussion

The results of this study demonstrate that for veterans enrolled in a residential SUD treatment program, supplementing their regular treatment programming with the TST-VR headset was associated with higher rates of successful treatment completion. Although this study was unable to include long-term data on recovery outcomes, prior research has demonstrated that completion of residential SUD treatment among veterans is associated with positive long-term outcomes when veterans are followed for five years, including increased aftercare attendance, lower risk of relapse, and lower mortality.^7^ Thus, the increased rates of treatment completion among individuals receiving the TST-VR headset may also be indicative of other longer-term positive health and recovery outcomes.

Although VR therapy has been used to treat mental health concerns among veterans, such as posttraumatic stress disorder,^17^ the use of VR therapy to treat SUDs has been limited. Per a recent systematic review on VR therapy for SUDs, VR therapy used for this purpose has primarily involved either cue exposure or craving induction therapy;^12^ though some VR therapy for SUDs has incorporated cognitive behavioral techniques, the basis for the intervention has still been cue exposure and craving induction.^18^ To our knowledge, TST-VR is the first integrative CBT with extensive CBT psychoeducation, as well as CBT activities specifically designed to target factors contributing to SUDs, that has been successfully implemented among residential patients. Further, although the feasibility of TST-VR has previously been reported,^13^ this study is the first to examine the association of extensive cognitive behavioral VR treatment for SUD and treatment outcomes.

In addition to its strengths, there are several limitations to this study. Notably, causal extrapolation of study results cannot be conclusively made at this time due to the self-selection of individuals who received a VR headset (i.e., individuals who accepted the VR headset may have already been more inclined to complete treatment due to increased treatment engagement motivation). Therefore, there are essentially two (nonmutually exclusive) possibilities for the observed relationship. As postulated, the immersive TST-VR experience may have improved treatment engagement and motivation. Alternatively, the decision of several Veterans to opt not to receive VR gear may have reflected low initial motivation for treatment, which would lend itself to reduced likelihood of treatment completion. The former possibility underscores the potential of VR for SUD psychoeducation in CBT programs. The latter would provide an important caveat—that VR’s impact on SUD treatment may not be fully understood and warrants further exploration and eventually a randomized controlled trial to assess the impact of TST-VR on treatment outcomes more definitively. It is also possible that the novel and entertaining nature of the TST-VR experience and its virtual wooded retreat may have been a factor in patient retention, as opposed to the specific content learned. Future research may consider implementing such a randomized controlled trial, in which patients in residential substance use treatment would be randomly assigned to receive TST-VR versus treatment as usual (or perhaps versus receiving a VR program with nonedifying but aesthetically similar virtual world content) and then followed to assess treatment completion, as well as longer-term treatment outcomes, such as abstinence and/or quality of life posttreatment. Additionally, due to the small residential treatment sample available, this study was limited in sample size. Future research should explore the use of TST-VR on treatment outcomes among other treatment programs with larger sample sizes.

## Abbreviations Used

AMA: against medical advice
CBT: cognitive behavioral therapy
SATP: substance abuse treatment program
SUD: substance use disorders
TST: Transcending Self Therapy
VAMC: Veterans Affairs Medical Center
VR: virtual reality
